# Western Pacific hydroclimate linked to global climate variability over the past two millennia

**DOI:** 10.1038/ncomms11719

**Published:** 2016-06-08

**Authors:** Michael L. Griffiths, Alena K. Kimbrough, Michael K. Gagan, Russell N. Drysdale, Julia E. Cole, Kathleen R. Johnson, Jian-Xin Zhao, Benjamin I. Cook, John C. Hellstrom, Wahyoe S. Hantoro

**Affiliations:** 1 Department of Environmental Science, William Paterson University, Wayne, New Jersey 07470, USA; 2 Research School of Earth Sciences, Australian National University, Canberra, Australian Capital Territory 0200, Australia; 3 School of Geography, University of Melbourne, Parkville, Victoria 3010, Australia; 4 EDYTEM, UMR CNRS 5204, Université de Savoie, 73376 Le Bourget du Lac, France; 5 Department of Geosciences, University of Arizona, Tucson, Arizona 85721, USA; 6 Department of Atmospheric Sciences, University of Arizona, Tucson, Arizona 85721, USA; 7 Department of Earth System Science, University of California, Irvine, California 92697-3100, USA; 8 School of Earth Sciences, University of Queensland, Brisbane, Queensland 4072, Australia; 9 NASA Goddard Institute for Space Studies, New York, New York 10025, USA; 10 School of Earth Sciences, The University of Melbourne, Parkville, Victoria 3010, Australia; 11 Research Center for Geotechnology, Indonesian Institute of Sciences, Bandung 40135, Indonesia

## Abstract

Interdecadal modes of tropical Pacific ocean-atmosphere circulation have a strong influence on global temperature, yet the extent to which these phenomena influence global climate on multicentury timescales is still poorly known. Here we present a 2,000-year, multiproxy reconstruction of western Pacific hydroclimate from two speleothem records for southeastern Indonesia. The composite record shows pronounced shifts in monsoon rainfall that are antiphased with precipitation records for East Asia and the central-eastern equatorial Pacific. These meridional and zonal patterns are best explained by a poleward expansion of the Australasian Intertropical Convergence Zone and weakening of the Pacific Walker circulation (PWC) between ∼1000 and 1500 CE Conversely, an equatorward contraction of the Intertropical Convergence Zone and strengthened PWC occurred between ∼1500 and 1900 CE. Our findings, together with climate model simulations, highlight the likelihood that century-scale variations in tropical Pacific climate modes can significantly modulate radiatively forced shifts in global temperature.

## Abstract (web summary)

Interdecadal modes of tropical Pacific ocean-atmosphere circulation have a strong influence on global temperature. Here, the authors present a 2000-year hydroclimate record from the Indo-Pacific, which suggests that century-scale variations in these modes are also linked with global temperature variability.

## Graphical abstract

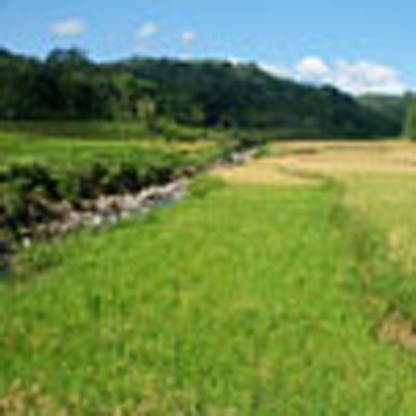

Deep atmospheric convection over the Indo-Pacific Warm Pool (IPWP) redistributes heat and moisture throughout the global climate system. The strength and location of IPWP convection is highly variable and strongly coupled to the Pacific Walker Circulation (PWC) and the associated modes of tropical climate variability, the El Niño-Southern Oscillation (ENSO) and the Interdecadal Pacific Oscillation. Changes in IPWP convection impact extra-tropical climate via meridional shifts in the average position of the intertropical convergence zone (ITCZ), which influences the intensity of the Asian Summer Monsoon and the Australian-Indonesian Summer Monsoon (AISM; Supplementary Figs 1 and 2). Meridional and zonal variations of the Indo-Pacific climate system have very different impacts on Earth's climate. Meridional shifts in the position of the ITCZ rapidly transmit hydroclimate perturbations across the hemispheres in response to changes in interhemispheric temperature gradients^1^. By contrast, zonal shifts in the PWC, which alter the trade wind fields and the location and strength of deep convection (both zonally and meridionally), extend the geographic reach of ENSO and contribute to global temperature changes^2^,^3^,^4^. Therefore, unravelling the history of deep atmospheric convection in the western equatorial Pacific region before instrumental measurements is critical for understanding the role of the tropics in modulating both regional and global climate variability.

Paleoclimate records spanning the last ∼2,000 years have highlighted the potential influence of Northern Hemisphere temperature changes in modulating Australasian atmospheric circulation via meridional shifts in the ITCZ^5^,^6^,^7^,^8^. However, the influence of zonal displacements in deep convection (associated with ENSO) in driving tropical climate is implicated by other studies^9^,^10^. As a result, there are different hypotheses regarding the precise drivers of tropical Pacific hydroclimate over the past millennium, and the relative importance of meridional forcing from the extratropics versus zonal ENSO-like shifts inherent to the tropics. To further examine these interactive climate systems, we have constructed a high-resolution multi-proxy hydroclimate record for southeast Indonesia spanning the last ∼2,000 years based on stalagmite oxygen (δ^18^O) and carbon (δ^13^C) isotopes, elemental ratios (Mg/Ca, Sr/Ca), and initial ^234^U/^238^U activity ratios. These data reveal substantial changes in AISM rainfall and deep atmospheric convection at the ascending limb of the PWC over the past two millennia. Comparison of our results with coupled general circulation model (CGCM) simulations reveal century-scale periods of divergence between the paleodata and model simulations, highlighting a deficiency in the models to capture the lower-frequency variability of the PWC. As such, the influence of the PWC to amplify the radiatively forced warming and cooling trends of the past millennium is limited in the models. These results have important implications for future projections of global temperatures because they show that state-of-the-art last millennium (AD 850–1850) and historical (AD 1850–2005) CGCM experiments may be underestimating the potential for low-frequency shifts in the tropical Pacific mean state to modulate Earth's climate.

## Results

### Cave setting

**Figure 1:**
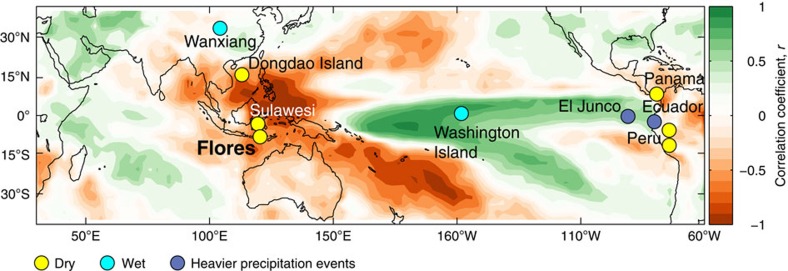
ENSO influence on tropical hydroclimate. Field correlation map of annual rainfall and east equatorial Pacific sea surface temperatures. Correlation coefficients (*r*) were calculated between GPCP v2.2 precipitation (at each 1° × 1° grid point) and HadISST1 NINO3.4 SSTs (1979–2010). The coloured dots show the location of our study site and other paleoclimate records mentioned in the text. Yellow indicates relatively dry conditions during the Medieval Climate Anomaly^5^,^6^,^9^,^44^,^46^,^48^ (950–1250 CE), whereas cyan/purple indicates relatively wet conditions^7^,^8^/intense precipitation events^10^,^47^.

The two stalagmites used in this study (LR06-B1 and LR06-B3) were collected from Liang Luar, an ∼1.7-km-long cave situated on the east Indonesian island of Flores (8° 32'N, 120° 26'E; 550 m above sea level; Fig. 1 and Supplementary Figs 1 and 2). Both specimens were located in a large chamber ∼600 m from the cave entrance and were actively growing at the time of collection in August 2006. Liang Luar resides close to the southerly limit of the austral summer ITCZ and most of the annual rainfall (∼70%) occurs during the AISM season (December to March)^11^.

### Stable isotope and trace element records

The hydroclimate record derived from stalagmites LR06-B1 and LR06-B3 is fixed in time by 35 uranium-thorium (U-Th) ages (Supplementary Figs 4–6) and spans the period 3 to 2005 CE (Common Era, Supplementary Table 1). All ages lie in stratigraphic order (within 2σ uncertainty) and have typical uncertainties of 1–3%. The age models for both records were first calculated using a Bayesian–Monte Carlo approach^12^ and then refined using the intra-site correlation age modelling programme (*iscam*)^13^ written in MATLAB (see the Methods for details). The age models give an average growth rate of ∼125–135 μm per year for both stalagmites.

**Figure 2:**
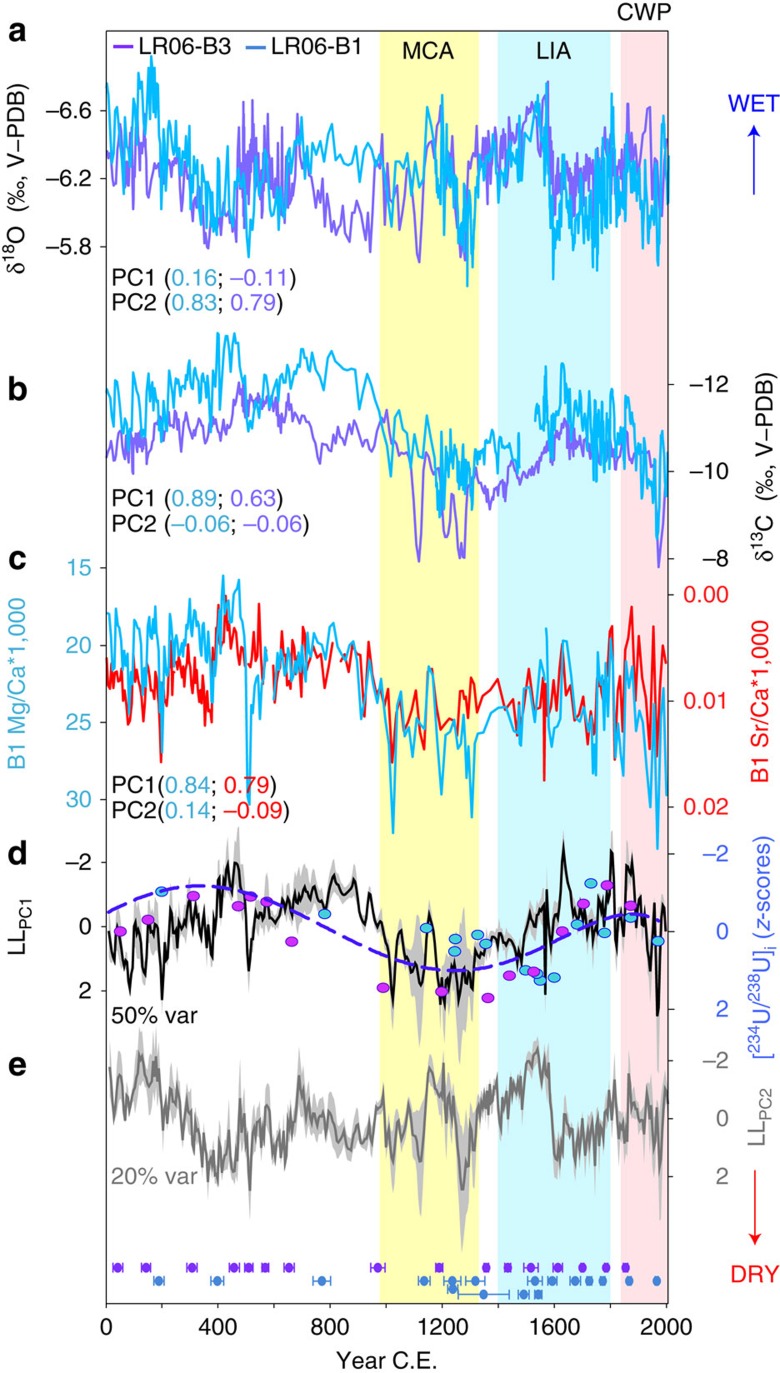
Multi-proxy reconstruction of east Indonesian hydroclimate. Comparison of stalagmites LR06-B3 and LR06-B1 (**a**) δ^18^O, (**b**) δ^13^C, (**c**) Mg/Ca (cyan) and Sr/Ca (red), (**d**) LL_PC1_ (black line) and [^234^U/^238^U]_i_ (purple dots: LR06-B3; blue dots: LR06-B1; dashed line: cubic spline fitted to the data) and (**e**) LL_PC2_. We interpret higher (lower) values in LL_PC1_ and [^234^U/^238^U]_i_ in **d** to reflect drier (wetter) conditions. Mg/Ca and Sr/Ca records were previously reported in Griffiths *et al*.^14^. The PC factor loadings for each time series of PC1 and PC2 are shown in parentheses. PC1 accounts for 50% of the variance and PC2 accounts for 20%. Colour-coded symbols along the bottom indicate U-Th ages (purple: LR06-B3; blue: LR06-B1) and their associated 2σ uncertainties. The grey shading in **d**,**e** indicates the 95% bootstrap confidence interval. Vertical bars indicate the approximate timing of the Medieval Climate Anomaly (MCA; yellow), Little Ice Age (LIA; blue) and Current Warm Period (CWP; pink) in Flores, defined by decreased AISM rainfall during the MCA and CWP, and increased AISM rainfall during the LIA.

The δ^18^O and δ^13^C records are based on 1,157 measurements performed on calcite powders micromilled along the central growth axes of LR06-B1 and LR06-B3. The Mg/Ca and Sr/Ca ratios in stalagmite LR06-B1, previously presented in Griffiths *et al*.^14^, were analysed on H_3_PO_4_ residues following calcite dissolution for stable isotope analysis. The strong degree of replication between the two stable isotope profiles (Fig. 2, Supplementary Fig. 7 and Supplementary Table 2), along with ‘Hendy' screening tests^15^ conducted on these specimens^11^, rules out significant disruption of the records by kinetic isotope fractionation effects.

The δ^18^O values for LR06-B1 and LR06-B3 range from −5.6 to −6.9‰ and average −6.2‰ between 1 and 2005 CE, whereas the δ^13^C values range from −7.8 to −13.4‰ and average −10.8‰. Broadly speaking, the δ^18^O and δ^13^C records display multidecadal- to centennial-scale oscillations with generally lower values from ∼500 to 900 CE and ∼1300 to 1600 CE, and generally higher values from ∼1000 to 1300 CE The Mg/Ca and Sr/Ca profiles show patterns similar to the stable isotopes over the last millennium, with generally higher values from ∼1000 to 1300 CE followed by a trend to lower values until ∼1600 CE Between ∼1600 CE and ∼2004 values have generally increased, signifying an overall reduction in rainfall over this period with the trend being most significant during the twentieth century.

### Interpretation of the stalagmite δ^18^O record

The seasonal pattern of monsoon rainfall at Liang Luar is reflected in the δ^18^O of precipitation (δ^18^O_p_), with summer rainfall (DJFM) ∼6–7‰ lighter than its winter monsoon (JJA) counterpart^11^. Hence, a change in the fraction of the year dominated by the summer monsoon (that is, change in seasonality), or a shift in monsoon rainfall amount in summer, should have a significant impact on the average annual δ^18^O value. The strong relationship between Indonesian rainfall amount and δ^18^O_p_ has been identified in modern isotope studies^11^ and CGCM simulations of modern^16^,^17^ and past^18^ hydroclimates in Indonesia. Model simulations of precipitation δ^18^O_p_ using the National Center for Environmental Prediction (NCEP)/National Center for Atmospheric Research (NCAR) nudged IsoGSM^17^ for the grid point closest to Liang Luar reveal a significant correlation with sea-surface temperatures (SSTs) over the NINO3.4 region and, to a lesser extent, in the western Indian Ocean; this pattern is similar to the observed pattern of rainfall amount at Liang Luar (Supplementary Fig. 3). Moreover, comparison of Liang Luar IsoGSM δ^18^O_p_ with observed rainfall amount, outgoing longwave radiation and zonal 850 mb winds reveals that δ^18^O_p_ is strongly influenced by western Pacific rainfall variability, which is governed by the strength of the AISM, the position of the ITCZ and ENSO (Supplementary Fig. 3).

Based on observed and modelled precipitation δ^18^O systematics, we interpret variations in the Flores speleothem δ^18^O records to primarily reflect regional rainfall amount. However, on relatively short timescales (centuries), other factors may affect stalagmite δ^18^O, such as changes in cave temperature (due to shifts in regional SSTs), the δ^18^O of surface seawater^5^, the proportion of summer- and winter-sourced moisture^11^ and multidecadal changes in the relationship between δ^18^O_p_ and ocean-atmosphere climate modes, such as ENSO^19^ and the Indian Ocean Zonal Mode^20^. In addition, mixing of meteoric water in the soil-karst overburden (∼50–100 m) above Liang Luar reduces the amplitude of the highest frequencies^21^ (for example, seasonal-annual variations), which likely varies with time. Therefore, some of the observed variability may be an artefact of filtering through the karst network. However, given the amount of cave recharge through the epikarst each monsoon season, it is likely that the δ^18^O of the cave drip-water represents decadal (if not multi-year) shifts in precipitation.

### A composite multi-proxy stalagmite record

To constrain the Liang Luar paleomonsoon record, we compare the stalagmite δ^18^O with the contemporaneous carbon isotope (δ^13^C), Mg/Ca and Sr/Ca ratios, which primarily reflect Liang Luar karst hydrology^14^,^22^. Recent geochemical modelling of the soil-karst system above Liang Luar demonstrated that the δ^13^C in the late Holocene section of stalagmite LR06-B1 was controlled by the degree of prior calcite precipitation (PCP)^22^ in the epikarst ‘upstream' of the stalagmite. Under this process, periods of reduced recharge result in partial dewatering of the karst fracture system overlying the cave, which causes a greater proportion of the CO_2_ degassing to occur before the drips reach the growing stalagmite^23^. The waters therefore become supersaturated in CaCO_3_ before their emergence in the cave, causing upstream (prior) calcite precipitation in fractures and on stalactite tips. In the case of δ^13^C, carbonate precipitation results in a higher stalagmite δ^13^C owing to the greater removal of ^12^CO_2_ during the degassing process^24^,^25^. The opposite occurs during wetter intervals^25^ (Fig. 2). Additional climate-related factors may have influenced the stalagmite δ^13^C, such as changes in the ratio of C3:C4 plants, soil respiration rates, contributions from atmospheric CO_2_ (refs 26, 27) and the relative contribution of bedrock carbon due to changes in open- versus closed-system dissolution^22^. However, these factors are all susceptible to hydrological changes and fluctuate in the same sense as PCP (that is, higher δ^13^C values indicate drier conditions). Therefore, regardless of their relative contributions to the carbon-isotope mass balance, shifts in stalagmite δ^13^C are inextricably linked to hydroclimate variations at this site.

Additional support for a PCP signal is provided by the pattern of Sr/Ca and Mg/Ca changes over the last 2,000 years (Supplementary Fig. 8). Covariation between these two ratios is diagnostic of a hydrologic control^23^,^28^: increases in both indicate drier conditions because the preferential loss of Ca^2+^ from solution (relative to both Mg^2+^ and Sr^2+^) in the percolation waters during PCP raises the Mg/Ca and Sr/Ca of the drip waters. This is transmitted to the stalagmite as higher Mg/Ca and Sr/Ca. During wetter intervals, PCP is either reduced or absent, leading to lower Mg/Ca and Sr/Ca in the stalagmite^23^. The significant correlation between δ^13^C and both trace element ratios provides even more compelling evidence of hydroclimate-driven changes in the degree of PCP over the last 2,000 years (Supplementary Fig. 8). It not only reinforces the similar findings recorded in Liang Luar for the entire Holocene^14^, but is in good agreement with previous studies of short-term (seasonal to annual)^26^ and long-term (century to orbital)^29^ hydroclimate change.

To effectively combine the climate proxies for stalagmites LR06-B1 and LR06-B3 into one composite hydroclimate record, we conducted an unrotated principal component (PC) analysis on the six individual stable isotope and trace element time series. The leading PC (from here referred to as ‘LL_PC1_') represents the dominant hydrologic signal embedded in the records^30^ (Fig. 2). The stalagmite δ^18^O does not significantly load on LL_PC1_ indicating that the processes important in altering precipitation δ^18^O (vapour transport, oceanic source and regional temperatures)^31^ may not strongly affect the amount of moisture at the cave site. These additional effects on δ^18^O_p_ are best captured in the second PC (Fig. 2). Therefore, at century timescales, δ^18^O_p_ probably reflects the over-riding influence of large-scale ocean-atmospheric processes that do not strongly affect the total amount of precipitation at the site^32^. These findings highlight the importance of utilizing multiple speleothem geochemical tracers to reconstruct tropical hydroclimate during the relatively stable background climate state of the last two millennia.

Additional evidence to support LL_PC1_ as a hydrologic proxy is provided by initial uranium isotope activity ratios ([^234^U/^238^U]_i_) in the Liang Luar stalagmites (Supplementary Table 1 and Fig. 2d). Holocene [^234^U/^238^U]_i_ ratios of LR06-B1 and LR06-B3 reflect the extent of dissolution related to the rate of groundwater movement in the epikarst of Liang Luar^14^,^30^. The [^234^U/^238^U]_i_ values increase during drier periods when relatively slow-moving percolation waters (in approximately chemical equilibrium with the host rock) preferentially remove ^234^U (produced by alpha recoil or preferential leaching and loosely bound in limestone bedrock)^33^. In contrast, during wetter periods, the more rapid traverse of the percolation water through the karst network can result in greater dissolution rates causing both U isotopes to be leached approximately equally from the host rock. This can lead to relatively low [^234^U/^238^U]_i_ values in a stalagmite. Comparison of the [^234^U/^238^U]_i_ values with LL_PC1_ reveals similar multicentury trends over the past 2,000 years.

A final confirmation that LL_PC1_ reflects a regional hydroclimate signal over the western Pacific is provided by the significant correlation with other paleohydrologic records from the region, including a seawater δ^18^O record^5^ (*r=*0.5, 50-year smoothed, significant at the 99% level) and a leaf wax δD record^6^ (*r=*0.28, 50-year smoothed, significant at the 95% level) from marine sediment cores drilled in the Makassar Strait immediately west of Sulawesi (Fig. 1).

### Meridional shifts in the Australasian ITCZ

**Figure 3:**
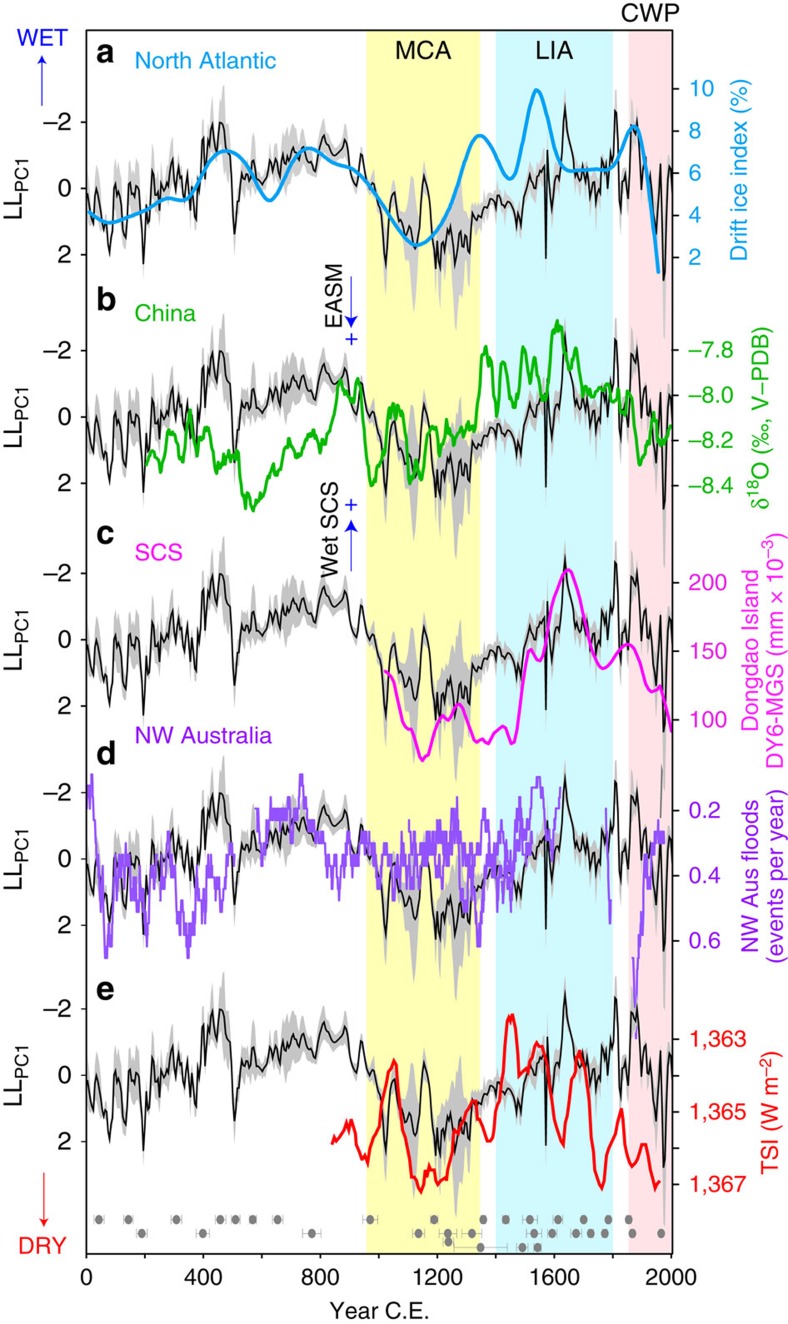
Tropical and high northern latitude teleconnections. (**a**) North Atlantic drift ice index^35^. (**b**) Stalagmite δ^18^O record from Wanxiang Cave, China^7^. (**c**) Mean grain size (MGS) of lake core DY6 from Dongdao Island in the South China Sea (SCS)^9^. (**d**) Northwest Australian flood events as recorded in a stalagmite from KNI-51 cave^42^. (**e**) Total solar irradiance reconstructed from cosmogenic nuclides^43^. The black line and grey shading in all panels shows the LL_PC1_ record and corresponding 95% bootstrap confidence interval. The *y* axis for the LL_PC1_ record is oriented to show a weaker AISM with warmer North Atlantic temperatures. Positions of the U-Th dates (with 2σ uncertainties) for both stalagmites are shown along the bottom. Vertical bars indicate the approximate timing of the MCA (yellow), LIA (blue) and CWP (pink) in Flores, defined by decreased AISM rainfall during the MCA and CWP, and increased AISM rainfall during the LIA.

The LL_PC1_ record exhibits pronounced century-scale excursions, with positive values (drier climate) at ∼0–400 CE, ∼1000–1400 CE and ∼1900–2004 CE, and negative excursions (wetter climate) at ∼400–1000 CE and ∼1400–1900 CE (Fig. 3). The dry period at around the turn of the last millennium coincides with the approximate timing of the Medieval Climate Anomaly (MCA; ∼950–1250 CE)^34^. This ∼400-year reduction in AISM rainfall begins to recover in strength at around 1300, and by 1500 is above average during the Little Ice Age (LIA). Maximum rainfall in Flores at ∼1600 CE, among the wettest periods of the past 2,000 years, is synchronous (within dating uncertainty) with peak cooling in the Northern Hemisphere^34^ and maximum ice discharge in the North Atlantic^35^ (Fig. 3a).

Comparison of the Flores LL_PC1_ record with subtropical paleomonsoon records from China^7^ (Fig. 3b) reveals synchronous (antiphased) multicentury fluctuations in monsoon rainfall across the hemispheres. The cross-equatorial antiphase relationship is consistent with both CGCMs^36^ and glacial paleoclimate records^37^,^38^, showing that cooling of the high northern latitudes increases the interhemispheric thermal gradient, displacing the Australasian ITCZ southward. However, zonal changes in the PWC are also known to strongly influence western Pacific hydroclimate^39^, and are thought to be a significant driver of low-latitude climate change on century scales^40^. Indeed, an in-phase relationship between LL_PC1_ and paleohydrologic records from the South China Sea^9^ (Fig. 3c) challenges the paradigm of regional north–south ITCZ migrations as the primary control of lower-frequency monsoon shifts in the western Pacific over the past millennium. Critically, a recent synthesis of paleohydrologic records for the Australasian monsoon region^41^, including a new northwest Australian flood record^42^ (Fig. 3d), demonstrated that, rather than moving southward during the LIA, the latitudinal range of monsoon-ITCZ migration probably contracted equatorward, perhaps in response to lower solar irradiance^43^ (Fig. 3e).

### Zonal teleconnections via the PWC

**Figure 4:**
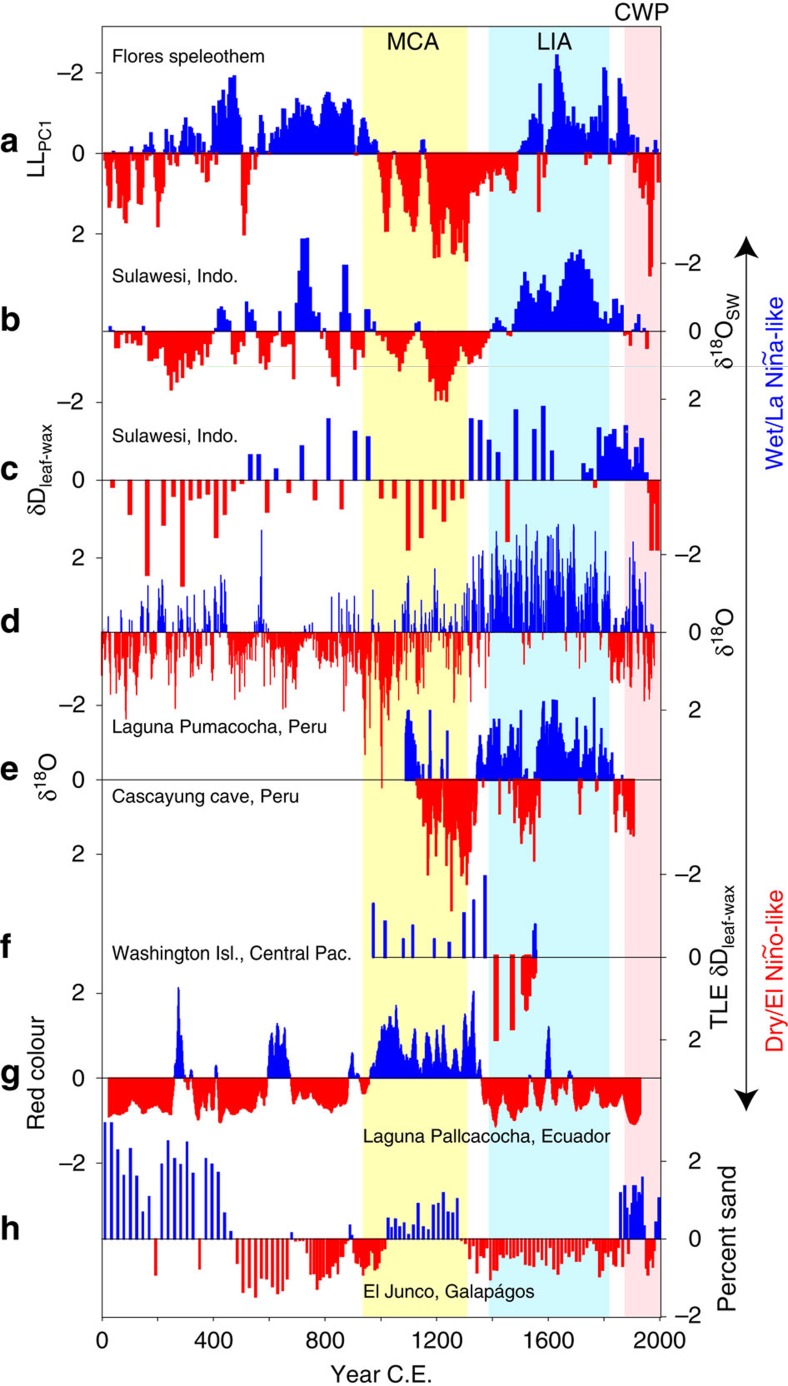
Hydroclimate records for the tropical western and eastern Pacific. (**a**) Flores LL_PC1_ record. (**b**) Marine foraminifera δ^18^O_sw_ (ref. 5) and (**c**) terrestrial δD_leaf-wax_ (ref. 6) records recovered from marine sediment cores located in the Makassar Strait on the Sulawesi margin. (**d**) δ^18^O of lake sediment calcite in Laguna Pumacocha in the central Peruvian Andes (proxy for the strength of the South American summer monsoon)^44^. (**e**) Speleothem δ^18^O record from Cascayunga cave in northeast Peru^46^. (**f**) δD_leaf-wax_ record from Washington Island in the central equatorial Pacific^8^. (**g**) Red-colour intensity from Laguna Pallcacocha, southern Ecuador^47^. (**h**) Percent sand in El Junco lake, Galápagos Islands^10^. For clarity, all records have been converted to standard (*z*) scores with blue indicating wetter conditions (**a**–**f**) or heavier precipitation events (**g**–**h**) and vice versa for red. Vertical bars indicate the approximate timing of the MCA (yellow), LIA (blue) and CWP (pink) in Flores.

Evidence for a potential link between the PWC and multi-century changes in western Pacific rainfall is provided by the inverse relationship between the AISM^5^,^6^ and rainfall in the central^8^-eastern^10^,^44^,^45^,^46^,^47^,^48^ equatorial Pacific (Fig. 4) over the past millennium. Specifically, the western Pacific and Peruvian Andes (Fig. 4a–e) were generally wetter during the LIA, whereas the central and eastern equatorial Pacific (EEP) experienced drier conditions (Fig. 4f) or reduced heavy precipitation events (Fig. 4g,h), similar to La Niña events today when the PWC strengthens (Fig. 1). Conversely, drier conditions in Indonesia and Peru (along with Panama^48^) during the MCA were matched by wetter conditions in the central and EEP, signifying a more ‘El Niño-like' mean state. Wavelet-transform analysis of the Flores LL_PC1_ shows significant multidecadal to centennial periodicities in the AISM record (Supplementary Fig. 8) that have also been identified in reconstructions of ENSO amplitude from North American tree rings^49^ and NINO3.4 SSTs^40^ (Supplementary Fig. 9).

**Figure 5:**
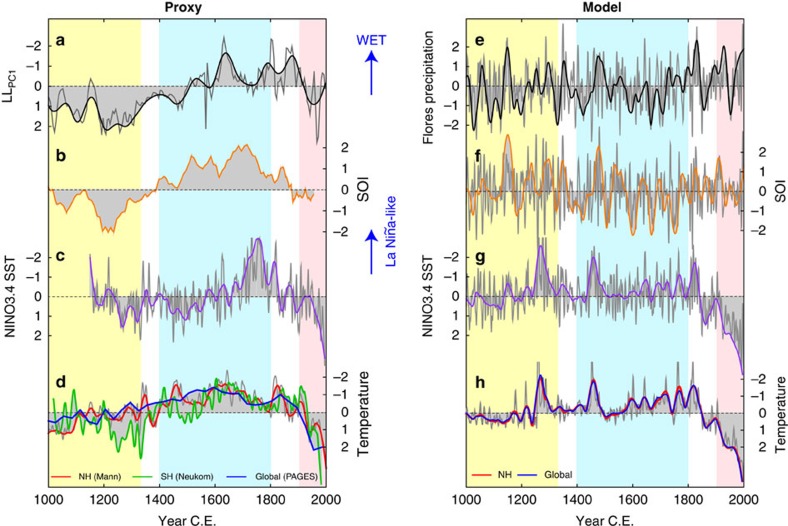
Western Pacific rainfall and ocean-atmosphere climate modes from paleoclimate records and climate model simulations. Panels **a**–**d** show paleoclimate records^34^,^40^,^50^,^59^,^60^ and **e**–**h** show CCSM4 LM+hist CGCM simulations. Comparison of Flores precipitation (**a**,**e**), SOI **(b**,**f**), NINO3.4 SSTs (**c**,**g**) and land temperatures (red: NH; blue: global; **d**,**h**). The records have been smoothed with a 50-year (thick coloured lines) and 7-year (thin grey lines) loess filter. All time series were converted to standard (*z*) scores to facilitate comparison of proxy reconstructions with model simulations. Vertical bars indicate the approximate timing of the MCA (yellow), LIA (blue) and CWP (pink) in Flores.

The multi-proxy record of eastern Indonesian hydroclimate presented here (LL_PC1_) lends support to a LIA characterized by a stronger PWC, whereas the LL_PC1_ shows drier conditions during the MCA, indicating a weaker PWC. These findings are compatible with the ITCZ expansion (LIA)/contraction (MCA) hypothesis of Yan *et al*.^41^ The temporal changes in zonal precipitation across the equatorial Pacific^5^,^6^,^8^,^9^,^10^,^40^ are also consistent with some terrestrial reconstructions of ENSO^40^,^45^,^50^ activity (Fig. 5), which show strengthening of the PWC during the LIA, and weakening during the MCA. These findings run counter to inferences from some SST-based ENSO reconstructions from the western^5^,^51^, central^52^ and EEP^53^, which suggest that the tropical Pacific was characterized by ‘La Niña-like' conditions during the MCA compared with the LIA. However, there is still no consensus on the marine paleo-ENSO signals; a recent reconstruction of EEP SSTs^54^ indicates that the MCA was marked by relatively warm SSTs in the EEP, contradicting the findings of earlier studies.

Theoretical and physical models of the PWC are also not entirely consistent within the context of Indo-Pacific climate change during the past millennium. Modern dynamical studies suggest that under warmer conditions, the PWC should weaken^55^,^56^, and at least one paleoclimate study supports the inference of a stronger PWC during the LIA^41^. On the other hand, the ‘ocean dynamical thermostat' mechanism^57^ supports a potential weakening of the PWC during the LIA. Modern observations do not yet distinguish the PWC's response to global warming. Yan *et al*.^41^ recently noted that there are numerous interacting components that may contribute to the discrepancies among paleoclimate records: (i) misinterpretation of SST or hydrological signals with respect to meridional versus zonal changes in ocean-atmosphere circulation; (ii) decoupling of ocean and atmospheric processes in response to different forcings over century time scales and (iii) the interacting influence of multidecadal climate modes (for example, Atlantic Multidecadal Oscillation) with ENSO, which are difficult to identify in the proxy records that originate from outside these system' centres of action.

### Palaeoclimate proxy-model comparisons

To explore the potential role of multidecadal- to century-scale changes in the PWC on global climate variability, we examined the relationship between Flores rainfall, NINO3.4 SSTs and the Southern Oscillation Index (SOI) with similar indices in CGCM simulations (Fig. 5). To do so, we employed the Paleoclimate Modelling Intercomparison Project Phase 3 (PMIP3) last millennium (LM; 850–1849 CE) and historical (1850–2005 CE) CGCM runs from the US NCAR CCSM4 (ref. 58) model. The CCSM4 model was employed because its simulation of the modern hydroclimate dynamics of east Indonesia compares well with observations. Importantly, model preindustrial control runs (500-year-long) accurately simulate the influence of ENSO on rainfall variability around Flores (Supplementary Fig. 10 and Supplementary Table 3). Although the association of SST with rainfall is somewhat stronger and more widespread in models compared with observations, the regional pattern of SST is consistent. The dominant influence of ENSO on east Indonesian rainfall is also apparent for the LM and historical (LM+hist; 850–2005 CE) simulations (Supplementary Table 3), although the correlation is not as strong as for the preindustrial control runs.

If variations in global temperatures over the LM were amplified by decadal to multidecadal variations in the tropical Pacific, as is the case during the instrumental period^2^,^3^,^4^, then model skill in the tropical Indo-Pacific region is clearly important for projecting future climate change. Therefore, we compared Flores precipitation, the SOI and NINO3.4 SSTs captured in the proxy records^40^,^50^ (Fig. 5a–c) with the same indices in the LM+hist CCSM4 simulations (Fig. 5e–g). In the LM+hist CGCM experiments, the variance in Pacific SST and Indonesian rainfall is concentrated at interannual/decadal time scales, whereas the paleoclimate record for Indonesia exhibits strongest variance at multidecadal to centennial periodicities (Supplementary Fig. 8). However, the coherent patterns of lower-frequency variability evident in the network of western Pacific hydroclimate records suggest that the data-model mismatch likely stems from model deficiencies with respect to century-scale variations in the PWC (Fig. 5b,c,f,g), particularly during the MCA and LIA. Consequently, the models may be underestimating the magnitude and duration of reconstructed global^59^ and hemispheric^34^,^60^ Medieval warming, and the persistent LIA cooling between ∼1400 and ∼1850 CE (Fig. 5d,h).

Our results highlight significant discrepancies between the proxy records and model simulations for the past millennium. Critically, these discrepancies coincide with century-scale anomalies in the strength of the PWC. We cannot rule out the possibility that some of the low-frequency Pacific variability was a forced response to variable solar intensity^36^ and changing teleconnections to higher latitudes^61^ that are not simulated by the models, or that non-climatic processes have influenced the proxies^62^. However, of particular importance is that the paleodata-model mismatch supports the possibility that unforced, low-frequency internal climate variability (that is difficult for models to simulate) was responsible for at least some of the global temperature change of the past millennium^62^,^63^.

## Discussion

The broad east–west antiphasing of Pacific hydroclimate during the past millennium, and clear connections with higher-latitude climates, suggests the deep tropics may have played a more active role in global climate change than previously assumed. Indeed, Northern and Southern Hemisphere temperature reconstructions^34^,^60^ (Fig. 5d) reveal symmetrical shifts in air-temperature over the past millennium (particularly during the LIA), suggesting that changes in the interhemispheric temperature gradient, and north–south shifts in the ITCZ, cannot explain the tropical precipitation variability evident in the proxy records. Instead, it is more likely that the LIA global cooling was initiated by a decline in solar irradiance (and lower atmospheric CO_2_ and potentially increased volcanic activity)^60^ and amplified by the strengthened PWC that occurred in parallel with a latitudinal contraction of the ITCZ^41^. Although it would be premature to suggest that LIA cooling was triggered by century-scale shifts in the tropical mean state, a stronger PWC during the LIA may have intensified broad-scale cooling already underway, and a weaker PWC during the MCA could have acted to warm the planet.

Our findings have significant implications for projections of decadal-scale changes in tropical atmospheric convection and global temperatures. For example, from the beginning of this century until recently, the tropical Pacific was locked into a negative Interdecadal Pacific Oscillation phase (that is, low-frequency La Niña-like pattern) in association with increased Walker and Hadley circulation winds and eastern Pacific cooling^2^,^3^. The La Niña-like pattern is thought to be a factor contributing to the recent so-called ‘warming hiatus'^2^,^3^ and earlier twentieth century cool and warm decades^4^. Therefore, our analysis of multicentury hydroclimate variability suggests that projections of tropical rainfall patterns, and global temperature extremes, will remain uncertain until paleoclimate records and models consistently capture the lower-frequency variability, and associated feedbacks, in the tropical Pacific.

## Methods

### ^230^Th dating and age models

A total of 35 pieces of calcite, each weighing ∼50–300 mg, were extracted from the central growth axis of stalagmites LR06-B1 and LR06-B3. Samples were removed using a carbide dental bur fitted to an air drill. ^230^Th/^234^U age determinations were conducted on both a thermal ionisation mass spectrometer and a multi-collector inductively coupled plasma mass spectrometer (MC-ICPMS; Supplementary Table 1 and Supplementary Figs 4 and 5). Thermal ionisation mass spectrometer analyses were carried out at the University of Queensland using a Fisons VG Sector 54–30 mass spectrometer equipped with a WARP filter and an ion counting Daly detector, as described by Yu *et al*.^64^ The MC-ICPMS U-Th analyses were conducted on two separate Nu-Instruments Nu Plasma mass spectrometers housed at the University of Melbourne and the University of Queensland. For a description of the MC-ICPMS methods employed at the University of Melbourne, refer to study by Hellstrom^65^. The methods employed at the University of Queensland are outlined in Zhou *et al*.^66^. All samples were corrected for initial ^230^Th using an initial ^230^Th/^232^Th value of 3.4±1.7. The initial ^230^Th/^232^Th value was determined by the methods described in the study by Hellstrom^65^. Corrected ages were calculated using half-lives specified in Cheng *et al*.^67^

The age models for LR06-B1 and LR06-B3 were first calculated using a Bayesian–Monte Carlo approach^12^, producing two δ^18^O time series that display a good level of coherence (Fig. 2 and Supplementary 7), despite the minor discrepancy between the two isotopic profiles during the period ∼700–1000 CE, which we attribute to slight differences in the resolution of U-Th dates through this interval (see Supplementary Figs 4 and 7 for details). Given this degree of replication, we refined the age models using *iscam*^13^ (that is, Intra-Site Correlation Age Modelling) with a prescribed point-wise linear interpolation scheme between adjacent U-Th ages. This novel age-model algorithm finds the best correlation between the proxy records for two (or more) neighbouring dated stalagmites (highest *r* value of 0.73 between LR06-B1 and LR06-B3) using a Monte Carlo approach (100,000 simulations using a 100-year smoothing), and then calculates the most probable age model for both records based on the calculated correlation coefficients. Because the two stalagmites were collected from the same cave chamber, we assume that the geochemical excursions preserved in the overlapping sections of both records reflect a real climate signal, and that minor discrepancies between the two are likely due to the uncertainties in the U-Th dates and slight differences in calcite stratigraphy. Significance levels were calculated against a red-noise background from 2,000 pairs of artificially simulated first-order autoregressive time series (AR1). The *iscam* age model provides a number of advantages to simple linear interpolation, which include: (i) quantitative assessment of whether the isotopic signals from multiple stalagmites have a common signal within age error; (ii) linking climatic proxies from multiple samples to construct a composite record and (iii) significant reduction of age uncertainty within overlapping time intervals through the enlargement of the signal-to-noise ratio.

### Stable isotope analysis

The oxygen (δ^18^O) and carbon (δ^13^C) isotope profiles for stalagmite LR06-B1 were established from 632 calcite powders micromilled along the central growth axis. The top 74 mm (that is, top of stalagmite down to a depth of 74 mm) of LR06-B1, which represents the ∼1172–2004 CE period of growth, was micromilled at 0.15 mm increments (∼1- to 4-year resolution). 30–70 μg of sample powders were analysed on a Thermo Finnigan Kiel IV carbonate device coupled with a Delta V Plus isotope ratio mass spectrometer at the University of California, Irvine (*n*=458 samples). Measurement precision (1σ) for the NBS-19 standard material was ±0.06‰ for δ^18^O and 0.03‰ for δ^13^C. The lower section of LR06-B1, representing ∼1–1172 CE period of growth (that is, depth between 74 and 246 mm from the top), was drilled at 1 mm increments (∼7- to 10-year resolution). The δ^18^O and δ^13^C for these lower-resolution samples was measured on ∼1 mg powders using a GV Instruments GV2003 continuous-flow isotope ratio mass spectrometer at the University of Newcastle, Australia (*n*=174 samples). The precision (1σ) of in-run measurements of the standard material (a Carrara Marble called New1) was ±0.08‰. These lower-resolution samples were previously reported in Griffiths *et al*.^11^

The δ^18^O and δ^13^C profiles for LR06-B3 were established from 525 calcite powders micromilled at 0.5 mm increments along the growth axis. The isotope measurements were performed at the University of Arizona on a Micromass Optima dual-inlet stable isotope ratio mass spectrometer with an automated carbonate preparation system. The precision (1σ) for in-run measurements (*n*=113) of laboratory standard material Luxor was ±0.07‰ for δ^18^O and δ^13^C. All results are expressed as the deviation in per mil (‰) between the sample and Vienna Peedee Belemnite standard using the delta notation.

### Trace element analysis

The Mg/Ca and Sr/Ca ratios in stalagmite LR06-B1 were analysed on H_3_PO_4_ residues after calcite dissolution for stable isotope analysis. The samples were analysed for Mg, Sr and Ca on a Varian Liberty 4,000 inductively coupled plasma atomic emission spectrometer. Molar concentrations of the elements were calculated from inductively coupled plasma atomic emission spectrometer intensities using four internal working standards of known concentration and a blank, with concentrations expressed as ratios to Ca. Acid blank corrections were applied to both sample and standard data before conversion of the data to ratios. The relative standard deviation of replicate standards of Ca, Mg and Sr was 1.5%, 1.6% and 2.4%, respectively. These data were previously published in Griffiths *et al*.^14^,^22^,^30^

### PC analysis

The speleothem hydroclimate record was constructed using the δ^18^O and δ^13^C for stalagmites LR06-B1 and LR06-B3, and trace elements (Mg/Ca, Sr/Ca) for LR06-B1. Due to the average resolution among all records being ∼5 years, we linearly interpolated each proxy record to a common 5-year temporal resolution before analysis. To extract the dominant climate signal embedded within the stable isotope and trace element records, we performed an unrotated PC analysis on the six profiles, where the leading PC (explains 50% of the variance) represents the dominant hydrologic signal common to all individual time series. Because other factors besides rainfall can affect the δ^18^O (for example, cave temperature, changes in ratio of summer versus winter moisture delivery) and δ^13^C (for example, above-cave vegetation, soil productivity, cave ventilation) and also the trace elements (for example, cave temperature, speleothem growth rate), this method has proven useful in isolating the dominant hydrologic signal from other potential influences^30^. However, the δ^18^O time series load weakly on PC1, and load much more strongly on PC2. This suggests that factors in addition to local rainfall amount likely influenced the speleothem δ^18^O. Confidence intervals (95%) for the PCs were calculated using bias-corrected and accelerated nonparametric bootstrap technique performed in MATLAB.

### General circulation modelling

To assess the long-term dynamical influence of the Walker circulation on IPWP convection, and NH and global temperatures, we used the PMIP3 LM (850–1849 CE) and historical (1850–2005 CE) CGCM runs from the US NCAR CCSM4 (ref. 58) model. The skill of the CGCMs in simulating the observed (that is, instrumental) ENSO influence on western Pacific hydroclimate was demonstrated by comparing field correlation maps between east Indonesian rainfall (annual precipitation averaged over a gridbox encompassing the area 12°–6° S and 110°–125° E) and SSTs in the historical simulations (1850–2005), with the same variables in the instrumental (1950–2010) records (Supplementary Fig. 10). In addition, comparison of east Indonesian rainfall with both NINO3.4 SSTs and the SOI in model preindustrial control runs (500-year-long) and the LM+hist (850–2005 CE) simulations reveals statistically significant *r* values (Supplementary Table 3), which again demonstrates the strength of the model in simulating the dominant climate mode in the western Pacific. Details of the model characteristics (that is, forcings, atmospheric levels, atmospheric resolution) for the LM and historical simulations, and preindustrial control runs, can be found in ref. 58.

### Data availability

Data from this paper are available at the NOAA World Data Center for Paleoclimatology (www.ncdc.noaa.gov/paleo).

## Additional information

**How to cite this article:** Griffiths, M. L. *et al*. Western Pacific hydroclimate linked to global climate variability over the past two millennia. *Nat. Commun.* 7:11719 doi: 10.1038/ncomms11719 (2016).

## Supplementary Material

Supplementary Information Supplementary Figures 1-10, Supplementary Tables 1-3 and Supplementary References
