# Supplementary material for: Western Pacific hydroclimate linked to global climate variability over the past two millennia
Source: Nat Commun. 2016 Jun 8;7:11719. doi: 10.1038/ncomms11719 (PMC4899856; doi:10.1038/ncomms11719)
Supplement: Supplementary Information — Supplementary Figures 1-10, Supplementary Tables 1-3 and Supplementary References [file ncomms11719-s1.pdf]

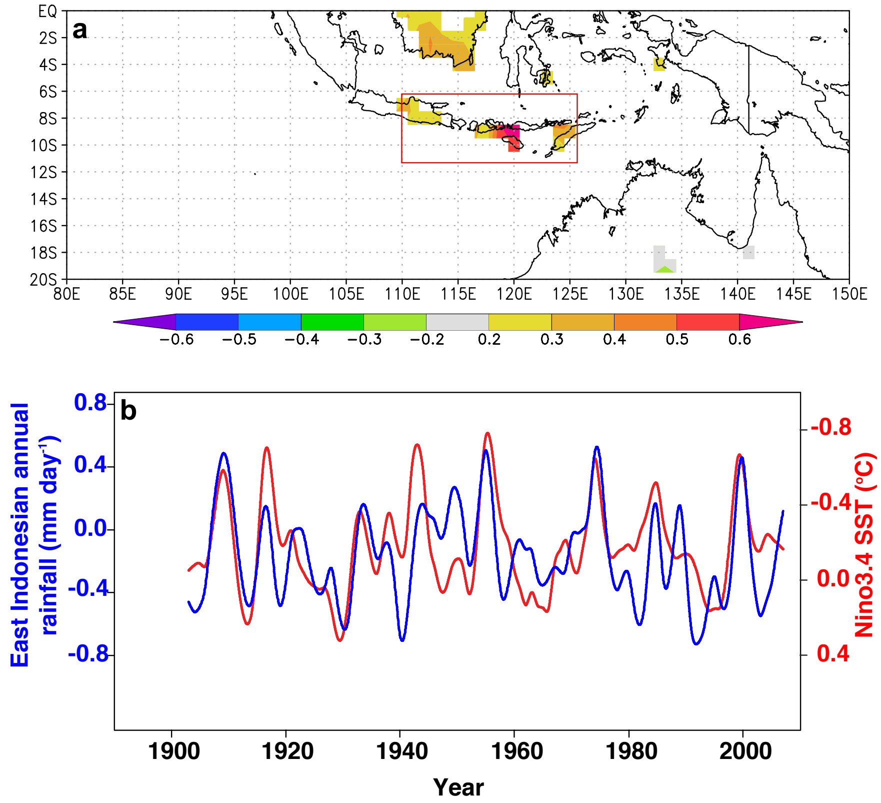

Supplementary Figure 1. a, Spatial correlation map between GPCC V6 (ref. 1) 1°x1° annual rainfall data at the Liang Luar cave site and rainfall at every other land-based grid-point on the map between 1901 and 2009. **b,** Comparison between east Indonesian rainfall (averaged over the area bounded by the red box in **a**) and Niño 3.4 HadI1SST data.

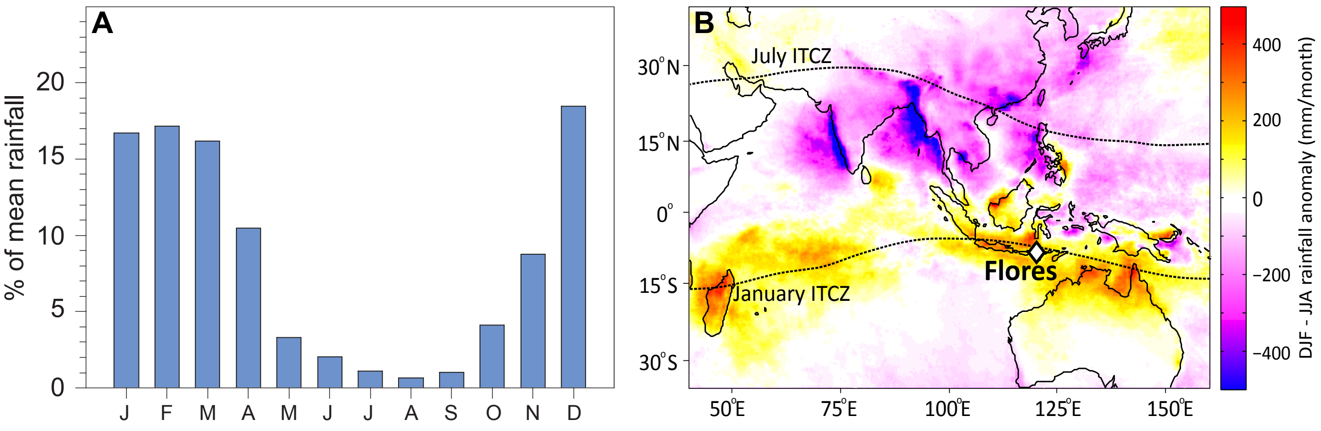

Supplementary Figure 2. a, Average monthly rainfall near the Liang Luar cave site between 1997 and 2007 derived from NASA's Tropical Rainfall Measuring Mission (TRMM) centered at 8.45°S and 120.47°E with 0.25° resolution. **b,** Seasonality index calculated as the difference between austral summer (DJF) and winter (JJA) precipitation using TRMM data.

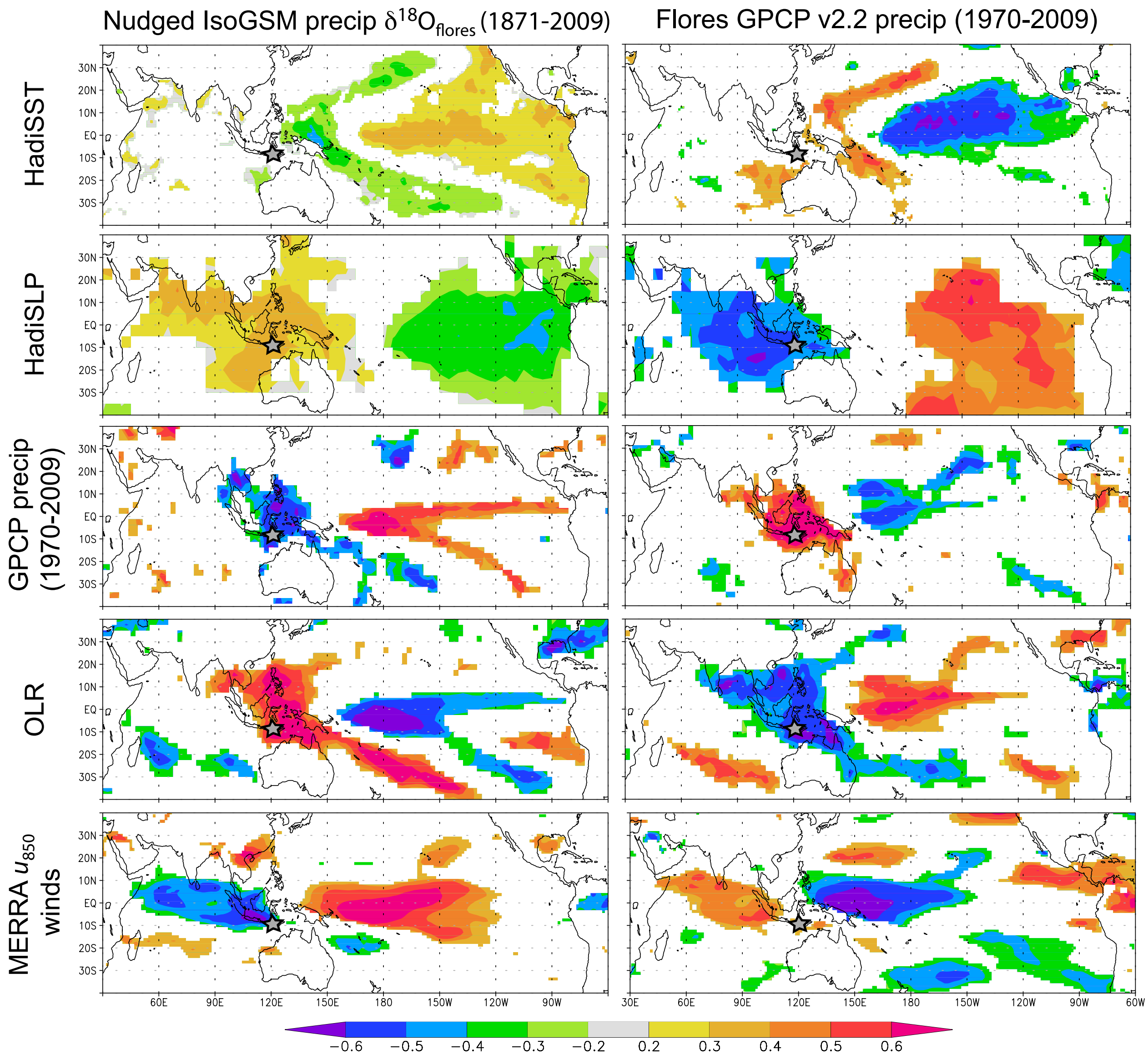

Supplementary Figure 3. Field correlation maps of Liang Luar (grey star) NCEP/NCAR nudged IsoGSM[2] precipitation amount-weighted $\delta^{18}O$ (left column) and GPCP v2.2 precipitation amount (right column) with SSTs (row 1), SLP (row 2), precipitation amount (row 3), outgoing longwave radiation (row 4), and NASA MERRA zonal 850 mb winds (row 5). Colors represent significant *r* values at the 90% level. It is evident from these plots that the relationship between the simulated amount-weighted $\delta^{18}O$ at Liang Luar and the various climatic parameters bears a strong resemblance to the relationship between the observed Liang Luar rainfall and the same parameters. These results provide strong support for the precipitation $\delta^{18}O$ at Liang Luar being a good proxy for regional moisture variability (rows 3-5), which is influenced by tropical Pacific and Indian Ocean SST gradients and SLP (rows 1,2).

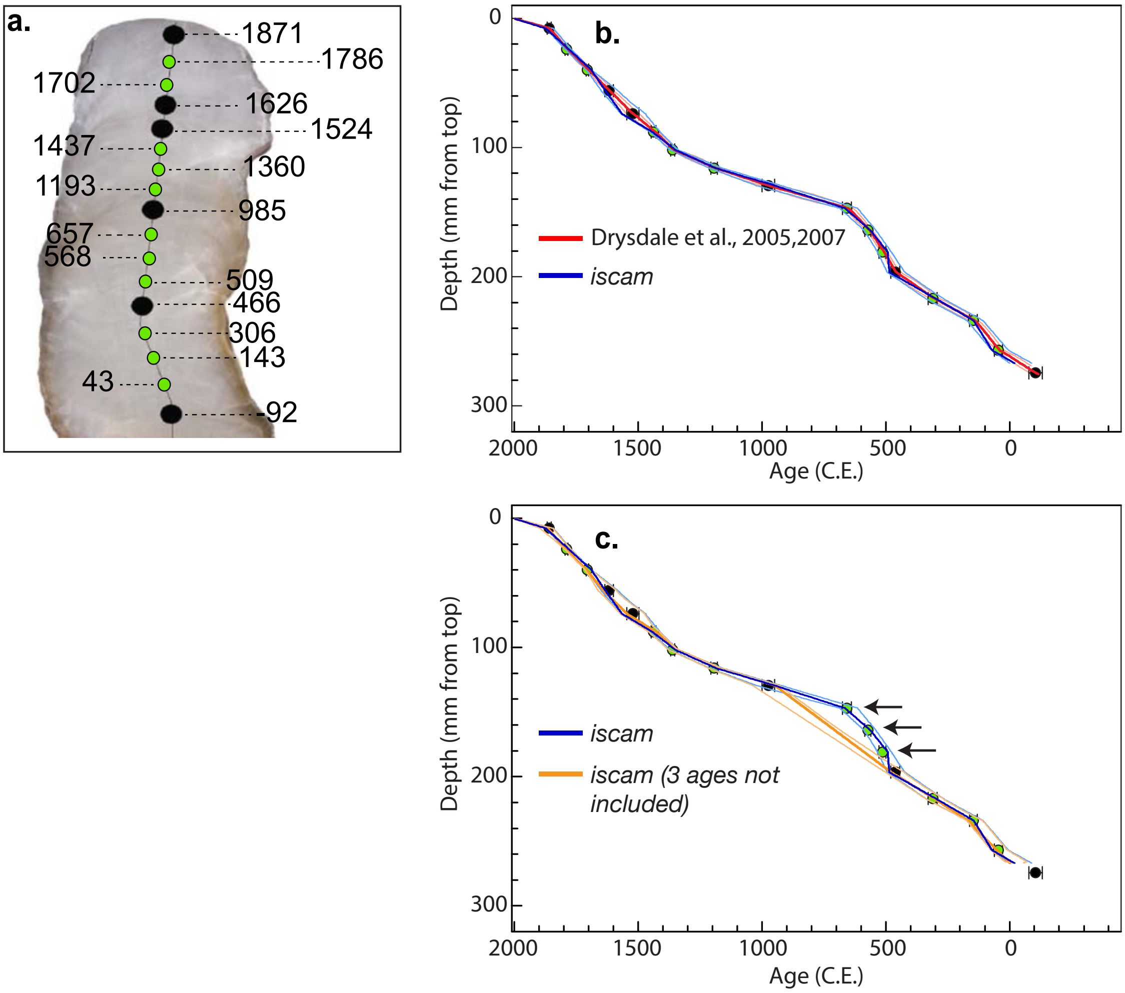

Supplementary Figure 4. Scanned image and *in situ* photo of stalagmite LR06-B3 and its associated age-depth models. **a,** Section of stalagmite LR06-B3 that was cut parallel to the growth axis. Green dots show the positions of new ^{230}Th dates measured to improve the age model previously reported in refs 3-6 (black dots). The ages are reported in “years C.E.” **b-c,** Age-depth plots for LR06-B3 using all ages shown in **a** (green and black dots). **b,** The red solid lines show the age models derived using methods described in Drysdale *et al.* (refs 7-8) whilst the blue lines show the age model calculated using the ISCAM algorithm (ref. 9). **c**, Same as **b,** but with three ages omitted (indicated by arrows) in the age ISCAM age model (orange line) indicated in Supplementary Fig. 7c. The color-coded lighter shaded lines show the calculated 95% uncertainty envelopes for the different age models.

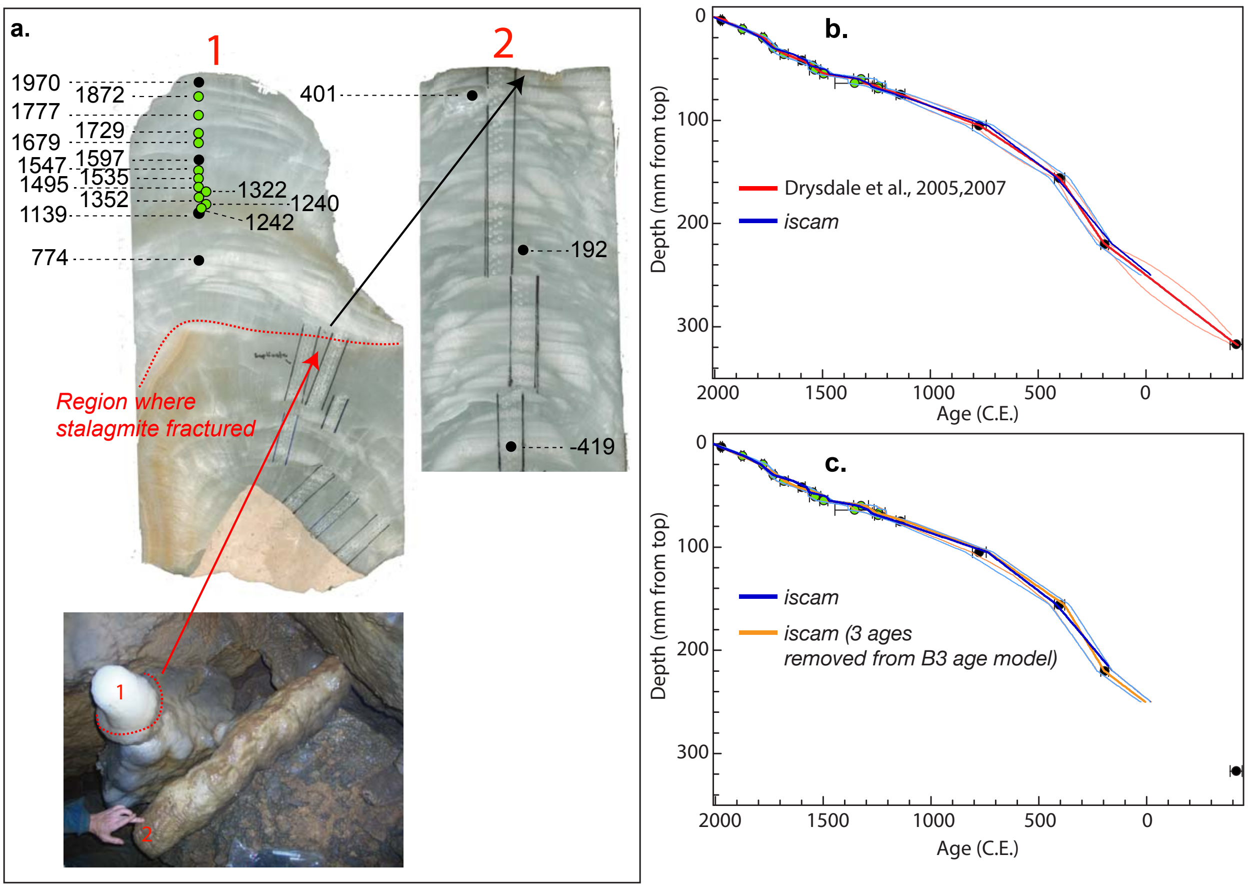

Supplementary Figure 5. Same as Supplementary Fig. 4 but for stalagmite LR06-B1.

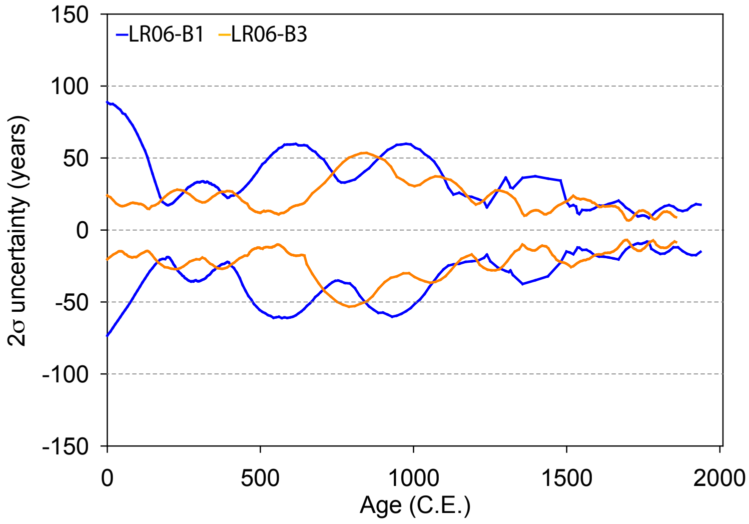

Supplementary Figure 6. Age uncertainty versus age plot for stalagmites LR06-B1 (blue) and LR06-B3 (orange) calculated by methods described in Drysdale *et al.* (refs 7-8).

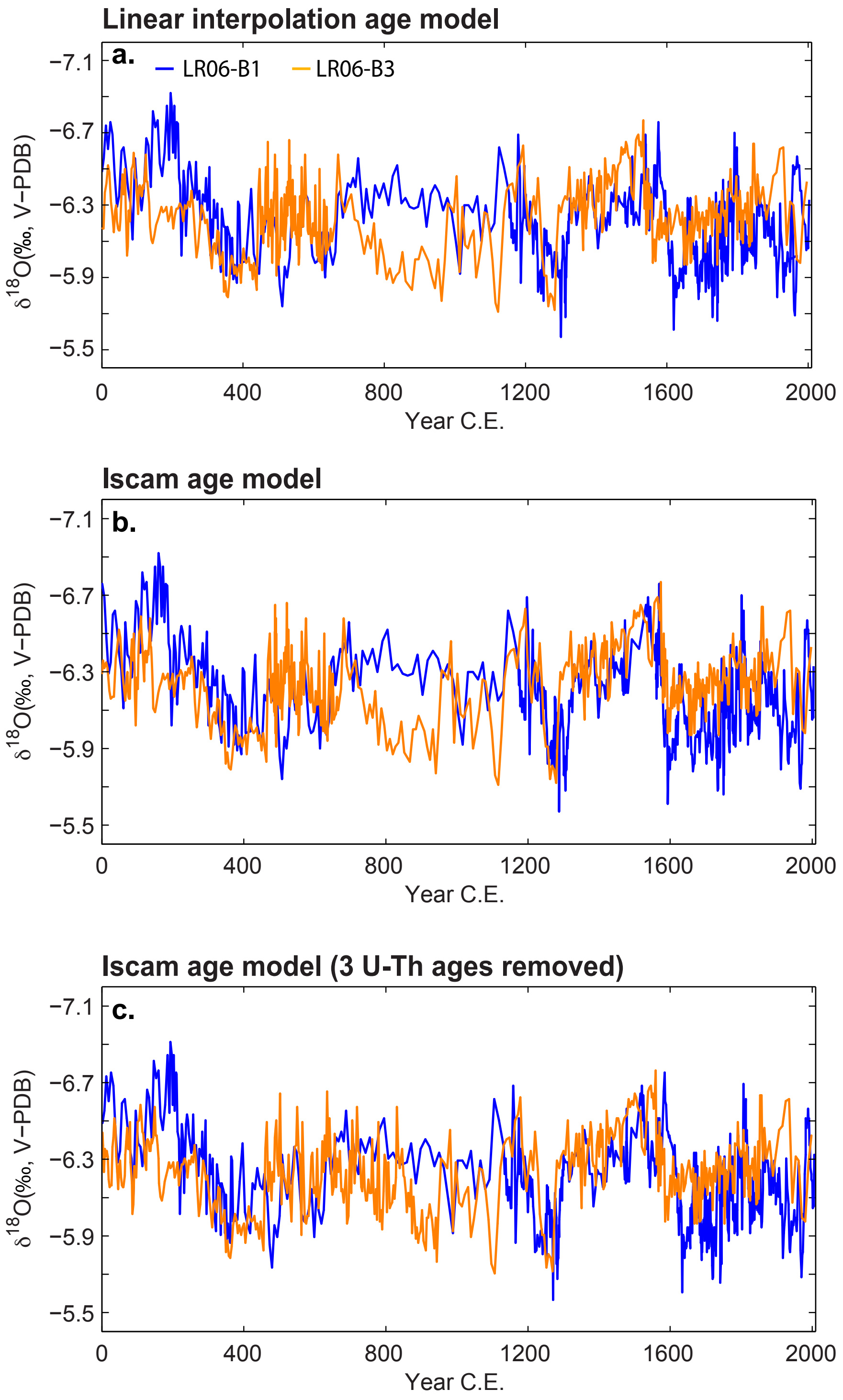

Supplementary Figure 7. Time series plots of $\delta^{18}O$ for stalagmites LR06-B1 (blue lines) and LR06-B3 (orange lines) for the age models derived from: **a,** Drysdale *et al.* (refs 7-8) and **b,** ISCAM[9]; **c**, Same as **b**, but with three ages removed (see arrows in Supplementary Fig. 4c) in the ISCAM age model. Removing these three ages from ISCAM age model increase the maximum (within uncertainty) correlation coefficient between the LR06-B1 and LR06-B3 $\delta^{18}O$ profiles from *r = 0.73* to *r = 0.77.* This higher *r* value is evident in the closer visual alignment of the two records between ~700-1000 C.E.

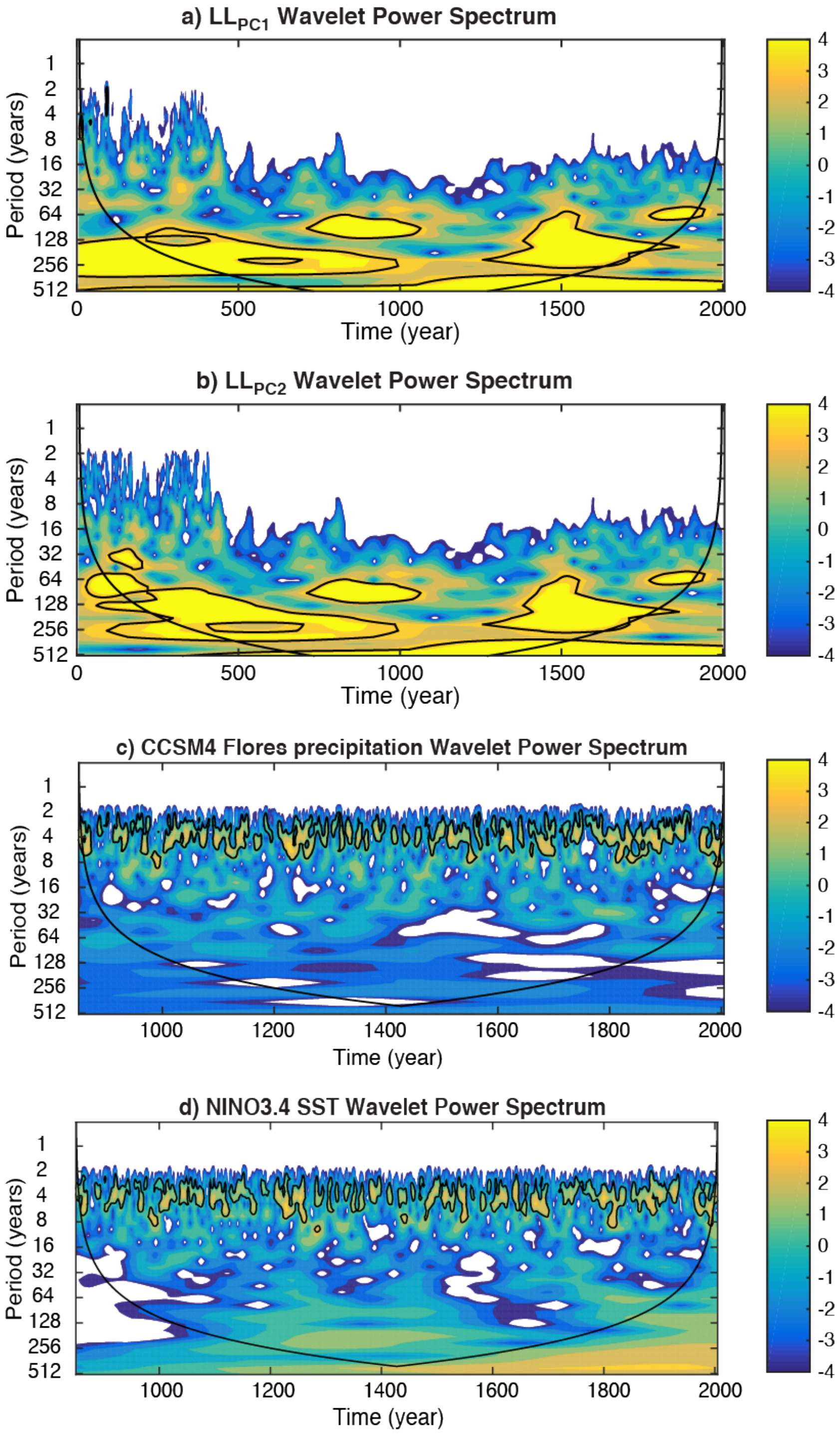

Supplementary Figure 8. Morlet wavelet power spectrum[10] for the **a**, LL_{PC1} and **b,** LL_{PC2} records compared with **c,** Flores rainfall and **d,** Niño 3.4 SSTs in the CCSM4 last millennial CGCM simulations. Black contours encompass values that exceed the 95% significance level using a red-noise background. Area below the black solid line towards the bottom of each figure is the cone of influence, where zero padding has reduced the variance. Results clearly show that the CCSM4 model lacks multi-decadal to centennial-scale variability in Indonesian monsoon rainfall, a reflection of the absent low-frequency Pacific SST variability.

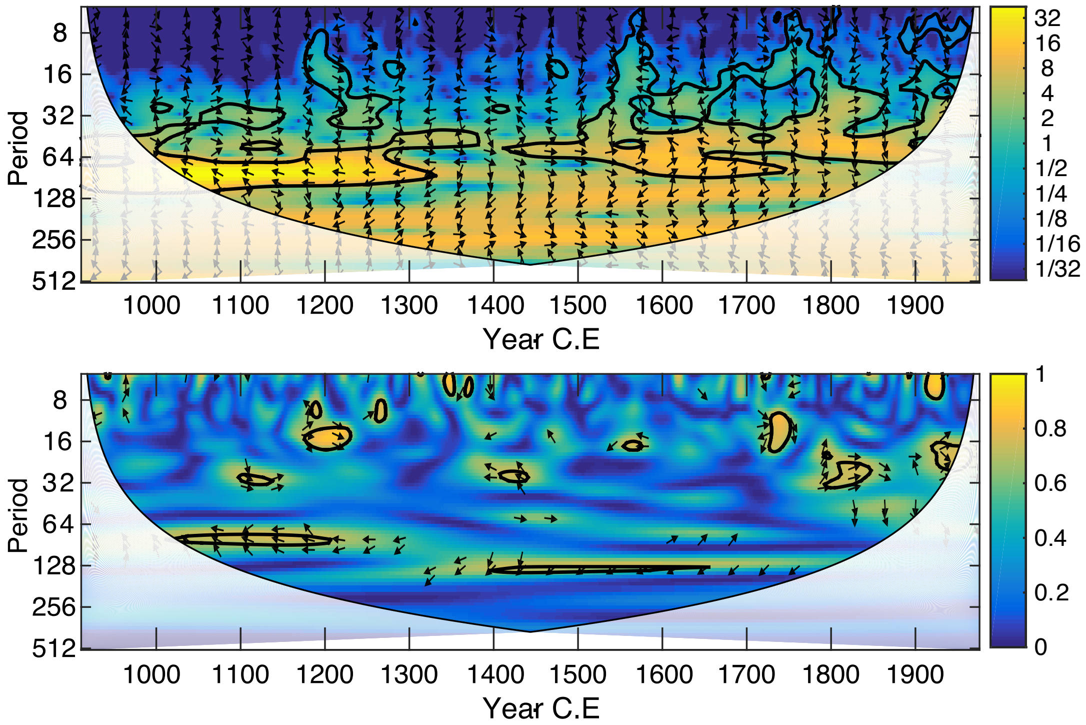

Supplementary Figure 9. Wavelet coherency analysis between LL_{PC1} and the tree ring North American Drought Atlas 21-yr biweight variance[11] calculated using the methods of Grinsted *et al.* (ref. 12). (Top) Cross-wavelet transform and (bottom) wavelet transform coherency (thought of as a correlation coefficient in time-scale space). Black lines encompass regions where the common power exceeds that expected from an identical low-order AR(1) process. Results show significant cross-wavelet power and high coherency at the mutlidecadal-centennial timescale.

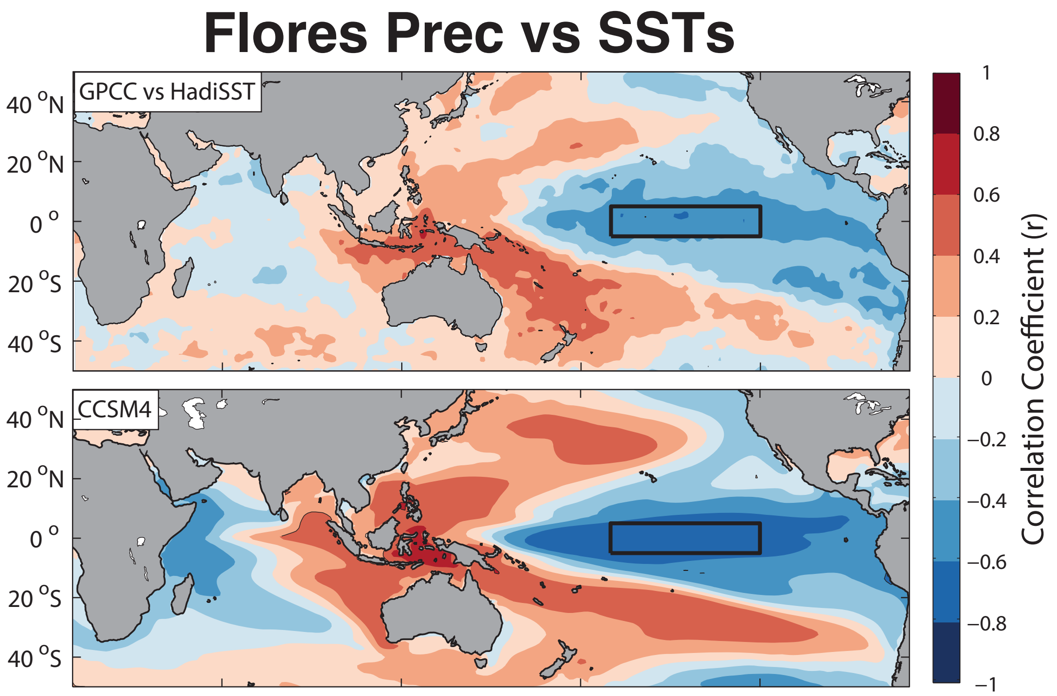

Supplementary Figure 10. Spatial correlation maps between instrumental and historical (1950-2005) CGCM simulations of Flores rainfall and SSTs. Annual Flores precipitation was averaged over the grid box encompassing the coordinates 12°-6° S and 110°-125° E, and then correlated with SSTs for the (top) instrumental (precipitation: GPCCv6[1]; SSTs: HadISST) and (bottom) NCAR CCSM4 CGCM simulations. Box denotes the Niño 3.4 region.

Supplementary Table 1. Summary of U-Th age data for stalagmites LR06-B1 and LR06-B3.

Sample I.D.	Core Depth (mm)	U (ppm)	[^{230}Th/^{238}U]	[^{234}U/^{238}U]	[^{230}Th/ ^{232}Th]	Uncorr. age (kyr BP)	Corr. age (kyr BP)	Corr. age (C.E)	Corr. initial [^{234}U/^{238}U]
B1/0003*	3	0.29	0.00061±0.00004	1.2526 ±0.0019	11	0.053 ±0.003	0.037 ±0.005	1970	1.2526 ±0.0019
B1/0012#	12	0.43	0.00173±0.00007	1.2436 ±0.0011	40	0.149 ±0.006	0.135 ±0.007	1872	1.2437 ±0.0011
B1/0020#	20	0.55	0.00276±0.00009	1.2492 ±0.0011	95	0.239 ±0.008	0.230 ±0.008	1777	1.2494 ±0.0011
B1/0030#	30	0.39	0.00343±0.00009	1.2306 ±0.0009	44	0.301 ±0.008	0.278 ±0.009	1729	1.2308 ±0.0009
B1/0036#	36	0.36	0.00458±0.00017	1.2461 ±0.0014	20	0.398 ±0.015	0.328 ±0.019	1679	1.2463 ±0.0015
B1/0042*	42	0.28	0.00492±0.00018	1.2660 ±0.0014	98	0.423 ±0.015	0.410 ±0.016	1597	1.2663 ±0.0014
B1/0047#	47	0.56	0.00546±0.00015	1.2670 ±0.0014	196	0.469 ±0.013	0.460 ±0.013	1547	1.2674 ±0.0014
B1/0051#	51	0.13	0.00674±0.00021	1.2644 ±0.0016	18	0.580 ±0.018	0.472 ±0.026	1535	1.2649 ±0.0016
B1/0055#	55	0.27	0.00691±0.00014	1.2633 ±0.0011	25	0.595 ±0.012	0.512 ± 0.019	1495	1.2637 ±0.0011
B1/0060#	60	0.51	0.00977±0.00018	1.2497 ±0.0015	17	0.854 ±0.016	0.685 ±0.034	1322	1.2502 ±0.0014
B1/0064#	64	0.20	0.01313±0.00027	1.2531 ±0.0015	8	1.146 ±0.024	0.655 ±0.091	1352	1.2536 ±0.0016
B1/0067#	67	0.22	0.01053±0.00017	1.2559 ±0.0013	21	0.916 ±0.015	0.767 ±0.030	1240	1.2564 ±0.0013
B1/0069#	69	0.53	0.00887±0.00021	1.2512 ±0.0015	363	0.774 ±0.018	0.765 ±0.018	1242	1.2518 ±0.0015
B1/0075*	75	0.23	0.01001±0.00024	1.2471 ±0.0015	289	0.876 ±0.021	0.868 ±0.021	1139	1.2477 ±0.0015
B1/0105*	105	35.48	0.01410±0.00036	1.2414 ±0.0012	349	1.242 ±0.032	1.233 ±0.032	774	1.2423 ±0.0012
B1/0156*	156	0.54	0.01801±0.00024	1.2058 ±0.0029	162	1.636 ±0.022	1.606 ±0.023	401	1.2067 ±0.0029
B1/0220*	220	0.36	0.02047±0.00020	1.2327 ±0.0019	592	1.820 ±0.018	1.815 ±0.018	192	1.2339 ±0.0019
B1/0317*	317	0.37	0.02776±0.00030	1.2522 ±0.0030	490	2.437 ±0.027	2.426 ±0.028	-419	1.2539 ±0.0030
B3/A-AB-a1*	7.75	0.34	0.00183±0.00009	1.2744 ±0.0014	25	0.156 ±0.008	0.135 ±0.013	1871	1.2745 ±0.0014
lr06b3-a-24	24	0.43	0.00261±0.00007	1.2617 ±0.0007	170	0.220 ±0.006	0.221 ±0.006	1786	1.2619 ±0.0007
lr06b3-a-40	40	0.44	0.00361±0.00007	1.2730 ±0.0005	242	0.304 ±0.005	0.303±0.006	1702	1.2732 ±0.0005
B3-101†	56	0.21	0.00470±0.00020	1.2901 ±0.0022	80	0.362 ±0.017	0.381 ±0.019	1626	1.2904 ±0.0022
B3-102†	74	0.18	0.00590±0.00030	1.3147 ±0.0026	235	0.475 ±0.025	0.483 ±0.025	1524	1.3151 ±0.0026
lr06b3-a-88	88	0.27	0.00696±0.00013	1.3170 ±0.0008	252	0.573 ±0.011	0.570 ±0.011	1437	1.3175 ±0.0008
lr06b3-a-102	102	0.39	0.00798±0.00010	1.3306 ±0.0007	269	0.650 ±0.08	0.647 ±0.009	1360	1.3312 ±0.0007
lr06b3-a-116	116	0.36	0.00998±0.00015	1.3267 ±0.0008	313	0.818 ±0.013	0.814 ±0.013	1193	1.3274 ±0.0008
B3-103†	129.5	0.25	0.01260±0.00030	1.3242 ±0.0022	172	1.000 ±0.025	1.022 ±0.027	985	1.3251 ±0.0022
lr06b3-a-147	147	0.32	0.01607±0.00022	1.2955 ±0.0008	426	1.357 ±0.019	1.350 ±0.019	657	1.2966 ±0.0008
lr06b3-a-164	164	0.30	0.01672±0.00013	1.2710 ±0.0008	1028	1.439 ±0.012	1.439 ±0.012	568	1.2721 ±0.0008
lr06b3-a-181	181	0.40	0.01735±0.00017	1.2678 ±0.0006	1473	1.498 ±0.015	1.498 ±0.015	509	1.2689 ±0.0006
B3-104†	196.5	0.31	0.01800±0.00020	1.2737 ±0.0023	482	1.530 ±0.018	1.541 ±0.019	466	1.2749 ±0.0023
lr06b3-b-217	217	0.37	0.01966±0.00021	1.2672 ±0.0008	2295	1.700 ±0.018	1.701 ±0.018	306	1.2685 ±0.0008
lr06b3-b-234	234	0.32	0.02181±0.00019	1.2816 ±0.0010	912	1.867 ±0.016	1.864 ±0.016	143	1.2831 ±0.0009

Sample I.D.	Core Depth (mm)	U (ppm)	[^{230}Th/^{238}U]	[^{234}U/^{238}U]	[^{230}Th/ ^{232}Th]	Uncorr. age (kyr BP)	Corr. age (kyr BP)	Corr. age (C.E)	Corr. initial [^{234}U/^{238}U]
lr06b3-b-257	257	0.34	0.02310±0.00020	1.2886 ±0.0013	930	1.968 ±0.017	1.964 ±0.017	43	1.2902 ±0.0013
B3-105†	274.4	0.12	0.02440±0.00030	1.2646 ±0.0022	294	2.075 ±0.027	2.099 ±0.029	-92	1.2662 ±0.0022

"U (ppm)" is the uranium concentration of the sample in parts per million. "Core Depth (mm)" is the mid-point distance from the top of the stalagmite for each U-series age. Activity ratios (in brackets) were determined by the methods described in Hellstrom (ref. 13). Corrected ^{230}Th ages were calculated using equation 1 of Hellstrom (ref. 14), assuming non-radiogenic ^{230}Th/^{232}Th=3.4±1.7 [calculated using the method of Hellstrom (ref. 14)], and half-lives specified in Cheng *et al.* (ref. 15).

†Dates determined by MC-ICPMS at the University of Melbourne, Australia.

*Date determined by TIMS at the University of Queensland, Australia. These data were published in Griffiths *et al.* (ref. 3).

$^{\#}$Date determined by MC-IPCMS at the University of Queensland, Australia.

Supplementary Table 2. Correlation matrix of the stable isotope and trace element data covering the past 2 ka. Higher resolution records were linearly interpolated to the lower resolution age-depth scale where appropriate prior to analysis.

	**B1-δ^{18}O**	**B1-δ^{13}C**	**B1-Mg/Ca**	**B1-Sr/Ca**	**B3-δ^{18}O**	**B3-δ^{13}C**
**B1-δ^{18}O**	–	0.46*	0.44*	0.10	0.33*	0.13
**B1-δ^{13}C**		–	0.68*	0.58*	-0.30*	0.66*
**B1-Mg/Ca**			–	0.55*	-0.12	0.46*
**B1-Sr/Ca**				–	-0.05	0.36*
**B3-δ^{18}O**					–	0.26*
**B3-δ^{13}C**						–

*Significant at the $P < 0.01$ level.

Supplementary Table 3. CCSM4 linear regression results for various comparisons between Flores precipitation (averaged over the 12°-6° S and 110°-125° E area), the Southern Oscillation Index (SOI), NINO3.4 SSTs, global air temperature (averaged over all land areas), NH air temperatures (averaged over all NH land areas), and NH SSTs in the preindustrial (PI) control runs (500-yr long) and the PMIP3 last millennium (850-1850 C.E.) plus historical (1850-2005 C.E.) simulations.

**CCSM4**	**PI Control** r^2	**p-value**	**Last millennium+historical** r^2	**p-value**
Flores Precip vs NINO3.4 SST	0.66	0.0000	0.59	0.0000
Flores Precip vs SOI	0.70	0.0000	0.66	0.0000
Globe Air-T vs NINO3.4 SST	0.27	0.0000	0.18	0.0000
Globe Air-T vs SOI	0.24	0.0000	0.03	0.0000
NH Air-T vs NINO3.4 SST	0.14	0.0000	0.13	0.0000
NH Air-T vs SOI	0.11	0.0000	0.01	0.0003
NH SST vs NINO3.4 SST	0.39	0.0000	0.20	0.0000
NH SST vs SOI	0.35	0.0000	0.05	0.0000

Supplementary References

1 Schneider, U. *et al.* GPCC Full Data Reanalysis Version 6.0 at 1.0°: Monthly Land-Surface Precipitation from Rain-Gauges built on GTS-based and Historic Data. doi:10.5676/DWD_GPCC/FD_M_V6_100 (2011).

2 Yoshimura, K., Kanamitsu, M., Noone, D. & Oki, T. Historical isotope simulation using Reanalysis atmospheric data. *J. Geophys. Res.-Atmos.* **113**, D19108, doi: 10.1029/2008jd010074 (2008).

3 Griffiths, M. L. *et al.* Increasing Australian-Indonesian monsoon rainfall linked to early Holocene sea-level rise. *Nature Geosci.* **2**, 636-639 (2009).

4 Griffiths, M. L. *et al.* Younger Dryas–Holocene temperature and rainfall history of southern Indonesia from $\delta^{18}O$ in speleothem calcite and fluid inclusions. *Earth Planet. Sci. Lett.* **295**, 30-36 (2010).

5 Griffiths, M. L. *et al.* Evidence for Holocene changes in Australian-Indonesian monsoon rainfall from stalagmite trace element and stable isotope ratios. *Earth Planet. Sci. Lett.* **292**, 27-38 (2010).

6 Griffiths, M. L. *et al.* Hydrological control of the dead-carbon fraction in a Holocene tropical speleothem. *Quat. Geochron.* **14**, 81-93 (2012).

7 Drysdale, R. N. *et al.* Stalagmite evidence for the precise timing of North Atlantic cold events during the early last glacial. *Geology* **35**, 77-80 (2007).

8 Drysdale, R. N., Zanchetta, G., Hellstrom, J. C., Fallick, A. E. & Zhao, J. x. Stalagmite evidence for the onset of the Last Interglacial in southern Europe at 129±1 ka. *Geophys. Res. Lett.* **32** L24708, doi:10.1029/2005GL024658 (2005).

9 Fohlmeister, J. A statistical approach to construct composite climate records of dated archives. *Quat. Geochron.* **14,** 48-56 (2012).

10 Torrence, C. & Compo, G. P. A practical guide to wavelet analysis. *Bull. Amer. Met. Soc.* **79**, 61-78 (1998).

11 Li, J. B. *et al.* Interdecadal modulation of El Nino amplitude during the past millennium. *Nature Clim. Change* **1**, 114-118 (2011).

12 Grinsted, A., Moore, J. C. & Jevrejeva, S. Application of the cross wavelet transform and wavelet coherence to geophysical time series. *Nonlinear processes in geophysics* **11**, 561-566 (2004).

13 Hellstrom, J. Rapid and accurate U/Th dating using parallel ion-counting multi-collector ICP-MS. *J. Anal. Atom. Spectrom.* **18**, 1346-1351 (2003).

14 Hellstrom, J. C. U–Th dating of speleothems with high initial ^{230}Th using stratigraphical constraint. *Quat. Geochron.* **1**, 289-295 (2006).

15 Cheng, H. *et al.* Improvements in ^{230}Th dating, ^{230}Th and ^{234}U half-life values, and U–Th isotopic measurements by multi-collector inductively coupled plasma mass spectrometry. *Earth Planet. Sci. Lett.* **371**, 82-91 (2013).
